# Rivastigmine: an open-label, observational study of safety and effectiveness in treating patients with Alzheimer's disease for up to 5 years

**DOI:** 10.1186/1471-2318-5-3

**Published:** 2005-01-19

**Authors:** Martin R Farlow, Mary L Lilly, for the ENA713 B352 Study Group

**Affiliations:** 1 Department of Neurology, Indiana University, Indianapolis, Indiana, USA; 2 Department of Neurology and Psychiatry, Indiana University, Indianapolis, Indiana, USA

## Abstract

**Background:**

Rivastigmine, a butyl- and acetylcholinesterase inhibitor, is approved for symptomatic treatment of Alzheimer's disease (AD). Data supporting the safety and efficacy of second-generation cholinesterase inhibitors, such as rivastigmine, are available for treatment up to 1 year, with limited data up to 2 1/2 years. The purpose of this report is to present safety and effectiveness data for rivastigmine therapy in patients with mild to moderately severe AD receiving treatment for up to 5 years.

**Methods:**

An observational approach was used to study 37 patients with originally mild to moderate AD receiving rivastigmine as a therapy for AD in an open-label extension (ENA713, B352 Study Group, 1998).

**Results:**

The initial trial demonstrated rivastigmine was well-tolerated and effective in terms of cognition, global functioning and activities of daily living. In this open label extension, high-dose rivastigmine therapy was safe and well tolerated over a 5-year period. Two thirds of the participants still enrolled at week 234 were in the original high-dose rivastigmine group during the double-blind phase, suggesting that early therapy may confer some benefit in delaying long-term progression of symptoms.

**Conclusions:**

Long-term cholinesterase inhibition therapy with rivastigmine was well tolerated, with no dropouts due to adverse effects past the initial titration period. Early initiation of treatment, with titration to high-dose therapy, may have an advantage in delaying progression of the illness.

## Background

Alzheimer's disease (AD) is the most common form of dementia affecting elderly people in the United States. Prevalence is 1% to 2% at age 65 years, but increases markedly to 35% or greater by age 85. Because of a demographic shift toward a more aged population, the percentage of affected individuals is rapidly increasing. This trend is expected to continue for the foreseeable future. Therefore, accurate and timely diagnosis and effective treatments are critical to optimal outcomes over the 8- to 10-year course of the illness [1].

Traditionally, a probable diagnosis of AD was accomplished by history, clinical examination, neuroimaging, and neuropsychological and laboratory testing to rule out treatable causes for the patient's symptoms and to differentiate AD from other possible causes of dementia [2,3]. Much effort has gone into defining risk factors for the development and progression of Alzheimer's dementia, as well as to identify biological markers for the disease. Clinical-demographic variables that are consistently associated with AD in prior studies include family history of AD, age, and Down's syndrome [1,3]. None of these variables has been demonstrated to affect the rate of disease progression or show any utility in defining subgroups that may be more amenable to therapy.

Currently, predominant symptoms of dementia are treated primarily with second-generation cholinesterase (ChE) inhibitors. These drugs have demonstrated efficacy, as measured by cognitive, behavioral, and functional outcomes, in randomized, placebo-controlled clinical trials, the majority of which have been of 6 months' duration [4-6]. In an open-label extension study of the cholinesterase inhibitor donepezil, Doody et al [7] concluded that donepezil was safe and effective for treating the symptoms of mild to moderate AD for up to 2 1/2 years. Cognitive, behavioral, and functional outcomes in patients treated with ChE inhibitors over the longer term are of great interest given the substantial social and economic implications of AD, which has a course that averages 8 to 10 years. Due to their relatively recent approval, however, longer-term data on the clinical benefits and/or limitations of ChE inhibitor therapy in AD patients is virtually nonexistent [8].

Rivastigmine's approval by the FDA in 2000 was supported by several pivotal trials, including a randomized US trial (ENA 713 B352)[5]. In this pivotal trial, 699 patients with mild to moderately severe AD were randomized to high dose rivastigmine (6–12 mg/day), low dose (1–4 mg/day) or placebo with a 7 week fixed dose-titration phase followed by a flexible dosing phase during weeks 8–26. Results of the 26-week open-label extension of this study found that at 52 weeks, patients originally treated with 6–12 mg/day rivastigmine had significantly better cognitive function than patients originally treated with placebo [9]. In this paper the authors present descriptive findings for a cohort of 37 patients who participated in the long-term open-label extension of the ENA713B352 rivastigmine trial.

Much work remains to be done to more definitively answer questions about when to start therapy, which patients are most likely to benefit, what constitutes clinically relevant beneficial effects over the longer term, and when these drugs are no longer clinically effective. Consideration should also be given to withdrawal of therapy. Findings presented in this article will add to the current limited dataset for long-term efficacy and outcomes with cholinesterase inhibitor therapy for persons with probable AD.

This report describes our experience in following the cohort of patients at our center with AD treated with the ChE inhibitor rivastigmine (a medication that inhibits both butyl- and acetylcholinesterase) as part of the ENA 713 B352 pivotal trial for a period up to 5 years.

## Methods

Data in this analysis came from a subgroup of 37 patients with originally mild- to moderate-stage (defined by a Mini-Mental State Examination [MMSE] score of 10 to 26) AD followed at a large Mid-Western university site in a 26-week, prospective, randomized, double-blind, placebo-controlled, parallel-group study of rivastigmine as therapy for AD conducted at 22 research sites across the United States (ENA713, B352 Study Group, 1998) [5].

Patients were enrolled according to previously described inclusion criteria [5]. Of note, this study allowed rather broad inclusion of AD patients with other comorbid illnesses; presumably this would allow this cohort to more closely mirror real-world populations. The study was conducted in accordance with ethical standards of the institutional committee on human experimentation and with the Helsinki Declaration of 1975, revised 1983. The initial study protocol was therefore reviewed in our center by the institutional review board and all patients or caregivers consented to inclusion based on appropriate informed consent. Additional consent was obtained for the open-label extension of the study.

### Cholinesterase inhibitor treatment

Immediately following the double-blind phase of the study, open-label rivastigmine was flexibly titrated over a 12-week time period to a maximum tolerated dosage of up to 6 mg BID. By the end of the 12 week titration 25 participants were on 4–6 mg. of rivastigmine and 11 participants had dropped from the study. One participant remained on a 2 mg. dose and dropped from the study between weeks 52 and 78.

### Assessment of treatment response

Outcomes measures included the Alzheimer's Disease Assessment Scale-cognitive subscale (ADAS-cog) and the Clinician's Interview-Based Impression of Change, with caregiver input (CIBIC-Plus). Ability to carry out activities of daily living (ADL) was assessed by the Progressive Deterioration Scale (PDS). Disease-staging measures included the Geriatric Deterioration Scale (GDS) and the MMSE. In the open-label phase of the study, efficacy evaluations were performed every 6 to 8 weeks for titration and early maintenance, and every 26 weeks for the next 5 to 6 years.

### Statistical analysis

These data are predominantly descriptive, with analyses including Kaplan- Meier survival plots when appropriate. All statistical analyses on our single center extension were performed at our center using SPSS. Subgroup analyses by initial treatment randomization were also performed.

## Results

### Demographics and population

Twenty-one Caucasian women and 11 Caucasian men participated in the open label extension of this pivotal study. Twenty-two participants reported a family history of AD. Selected demographic and baseline characteristics of the subsample are presented in Table I. Of the 32 patients, 11 were originally randomized to the high dose (6–12 mg/day) group, 10 to the low dose group, and 11 to placebo. Five of the patients in the cohort chose not to participate in the extension.

**Table 1:** Selected baseline and demographic characteristics (N = 32).

Characteristic	Mean (SD)
Age (y)	71.8 (7.5)
Length of symptoms (mo)*	29.6 (18.3)
Baseline MMSE	21.3 (4.1)
Baseline GDS	3.7 (0.78)

MMSE = Mini-Mental Status Examination; GDS = Geriatric Deterioration Scale.

*Length of symptoms prior to enrollment in the double-blind study.

**Figure 1:**
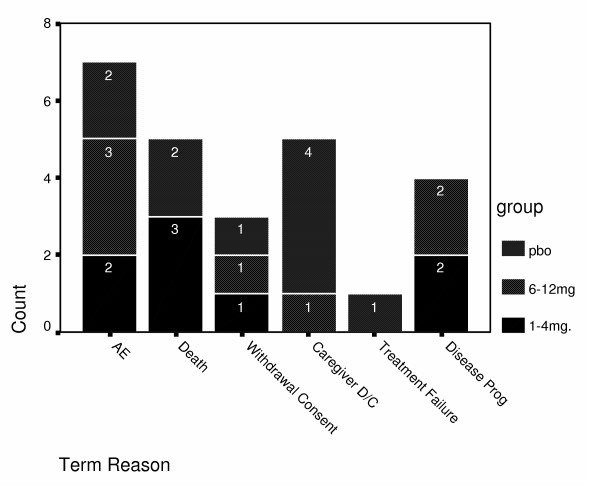
Reason for termination stratified by original group.

A total of 25 patients during the 5-year term eventually withdrew from the study. Reasons for termination, stratified by original group during the double-blind phase, are summarized in Figure 1. The most frequent reason for termination of participants initially randomized to the high-dose group was the lack of availability of free rivastigmine following FDA approval, which had been provided at pre-launch at no charge as part of the clinical study. It is of interest that no deaths occurred in the group initially randomized to high-dose rivastigmine during the initial double-blind, placebo-controlled trial. Furthermore, disease severity, as measured by the GDS, was greater in the original high-dose group (mean = 4.0; median = 4.0) as compared with the low-dose (mean = 3.7; median = 3.5) and placebo groups (mean = 3.4, median = 3.0). A total of 8 terminations were due to withdrawal of consent (n = 3) or caregiver discontinuation (n = 5). Five of the 8 were in the original placebo group. Seven patients withdrew because of adverse events; 2 each in the original low-dose and placebo groups (57%) and 3 in the original high-dose group (43%). Treatment failure was cited as the reason for termination of one 69-year-old man in the original placebo group. His baseline MMSE score was 21, and his final MMSE score at week 26 was 17. No other patients withdrew as a result of treatment failure.

Of the 4 patients who withdrew from the study due to disease progression, 3 were men between the ages of 57 and 64 years. The 57-year-old man was in the original high-dose group, with a baseline MMSE score of 16 and an MMSE score of 0 at the 234-week data collection. The other two male participants were aged 62 and 64 years with baseline MMSE scores of 22 and 20, respectively. The 62-year-old man in the original low-dose group withdrew at week 104 with an MMSE score of 9; the 64-year old man in the original high-dose group withdrew at week 156 with an MMSE score of 11. Interestingly, the 76-year-old woman in the original low-dose group, with a baseline MMSE score of 22, withdrew after the 26-week data collection (MMSE = 21).

### Quantitative (objective) analysis

**Figure 2:**
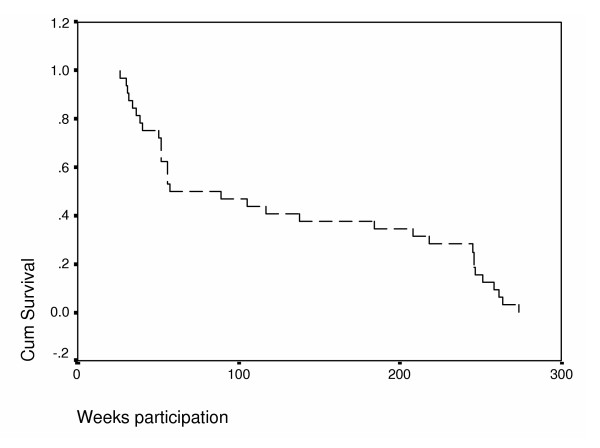
Kaplan-Meier analysis of time to dropout from week 26 to week 234

The Kaplan-Meier survival analysis, shown in Figure 2, illustrates time to dropout from week 26 to week 234. The survival curve reveals a relatively steep decline in participation in the first 9 months of open-label extension, mostly related to adverse events (AEs), since almost all AEs causing discontinuation occurred during this phase, followed by a "flattening" of the curve. Fourteen participants were still taking high-dose rivastigmine at 2 years, 12 participants at 3 years, and 10 participants at 4 years.

Of the subjects starting open-label rivastigmine, 25% were still participating at week 234. Of the 8 subjects still participating, 5 were female and only 1 reported a family history of AD. Interestingly, this group was characterized by a broad age range (57–85 years), a broad range of baseline MMSE scores (15–26), and by the fact that 5 of the 8 remaining participants at week 234 were in the original high-dose rivastigmine group.

For the end point defined as a 5-point drop from the baseline MMSE score, the group mean was 94 weeks. However, an age-related observation was noted such that the mean time to a 5-point drop from baseline MMSE for the ≤ 70 age group was 67 weeks as compared with 122 weeks for the >70 years age group. A similar trend was observed for scores on the ADAS-cog. For the group as a whole, mean time to a 4-point deterioration on the ADAS-cog was 84 weeks; with a mean of 70 weeks for the younger group and 104 weeks for the older group. [In the original study, mean age across the 22 research sites was 74.5 years; mean age for participants in this report was 71.8 years]

Figures 4 through 8 summarize mean scores for the ADAS-cog, CIBIC, GDS, PDS, and MMSE from week 26 through week 234. As expected, mean values for cognitive and functional status decline over time; however, significant within subject variability is present.

**Figure 3:**
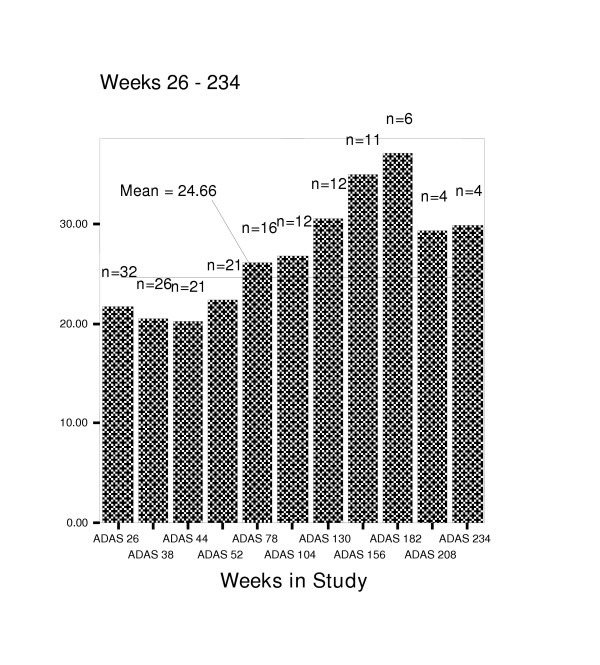
Mean scores on the Alzheimer's Disease Assessment Scale-cognitive subscale (ADAS-cog) for weeks 26–234.

### Qualitative analysis

When evaluating qualitative data, it is important to examine particular patients in terms of the data collected and treatment response at end of study. Data for the youngest man and the elderly woman, considered side-by-side, seem counterintuitive. Part of the explanation, however, may rest with the caregivers' experiences with and beliefs about the patients. This information can be accessed by reviewing the "symptoms most troubling" item from the CIBIC-Plus. This item asks, "With respect to the above symptoms, which are the biggest problems for you and/or other caregivers?" The spouse of the 57-year-old man reported that the "symptoms most troubling" for her were the patient's "selective difficulties and his attitude problem" (week 104, MMSE = 11) and that the patient "doesn't try" (week 130, MMSE = 7). These comments suggest a lack of acceptance of the patient's diagnosis and a lack of belief regarding the patient's documented decline that could explain continued clinic visits until, at week 234, the patient's MMSE score was 0. In contrast, the caregiver for the 76-year-old woman cited the fact that the patient "doesn't want to leave the house" (week 12) and the patient's anxiety about coming to clinic appointments (week 26) as the "most troubling" symptoms. These comments suggest that the caregiver was encountering patient resistance and observing patient distress, which were exacerbated by efforts to participate in the study. Therefore, further clinic visits were declined. Retrospectively, it is impossible to determine how the caregiver's beliefs and experience with the patient affect perceptions about disease status, treatment response, and decisions to continue or terminate treatment. These are interesting hypothesis-generating observations that should be explored in future investigations.

## Discussion

Limited data are available on the tolerability and effectiveness of cholinesterase inhibitor therapy for periods up to 3 years [4-7], but nothing has been reported in the literature concerning the percentages of patients remaining on therapy and its effect beyond this time point. This study adds to the limited long term data on patients treated with high-dose rivastigmine therapy by reporting descriptive data for up to 5 years. Findings presented in this report indicate that, in this sample of patients, high-dose rivastigmine therapy was well tolerated over a 5-year period.

Of note, approximately two thirds of the participants still enrolled at week 234 were in the original high-dose rivastigmine group during the double-blind phase; this finding suggests that early therapy with rivastigmine may confer some benefit in delaying long-term progression of symptoms, as has been previously suggested by analysis of the combined 26 weeks of double-blind and first 26 weeks of open-label data from the B352 US trial [9]. Throughout the initial 26-week double-blind portion, patients receiving placebo steadily deteriorated, while those treated with high-dose rivastigmine were able to maintain their baseline level of performance on the ADAS-Cog [5]. This approximated a delayed-start design for the open-label portion, which demonstrated that patients who started rivastigmine late never "caught up" with patients who had been on high-dose rivastigmine from the beginning of the trial. This suggests a disease-progression-delaying effect of the drug, which may allow this population to maintain their autonomy for a longer period of time. However, it is important to emphasize the limitations of this data; the analysis was retrospective, the sample was small, there were significant numbers of drop-outs, and the availability of free rivastigmine ceased with FDA approval which occurred near to the end of the study

## Conclusions

In summary, long-term therapy with the ChE inhibitor rivastigmine was well tolerated with no dropouts due to adverse effects past the first 9 months of the open-label extension. In contrast to the generally reported community experience of relatively brief duration of therapy with ChE inhibitors in AD, 25% of patients in this study were still taking rivastigmine by the end of 5 years. Of interest is the multifactorial nature of reasons for treatment discontinuation. Only 1 of these patients withdrew from the study because of perceived treatment failure over the 5-year period. Disease progression accounted for the withdrawal of 4 patients.

These results are informative both for the duration of treatment, and because the majority of the patients who continued treatment during this 5 year period were from the group originally titrated up to a high dose during the initial double-blind phase. Given that the sample size prohibits significance testing, these data suggest that rivastigmine therapy may be sustained over long periods of time in a significant percentage of patients with AD, and that high-dose therapy, particularly when begun early, may have an advantage in delaying the progression of the illness.

## Competing interests

MRF has received grant support and has served as consultant and received honoraria from Novartis Pharmaceuticals Corporation. MLL has no financial interest to declare.

## Authors' contributions

MF carried out the original clinical trial, conceived of the design for the observational study, and participated in the drafting and revisions of the manuscript. ML participated in the design of the observational study, performed statistical analysis, and participated in the drafting and revisions of the manuscript. Both authors read and approved the final manuscript.

**Figure 4:**
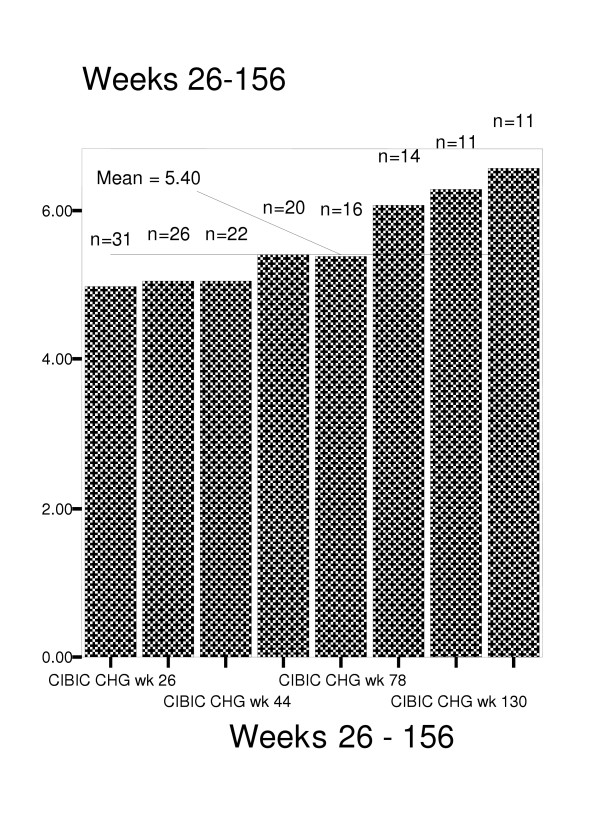
Change in Clinician's Interview-Based Impression of Change for weeks 26–56.

**Figure 5:**
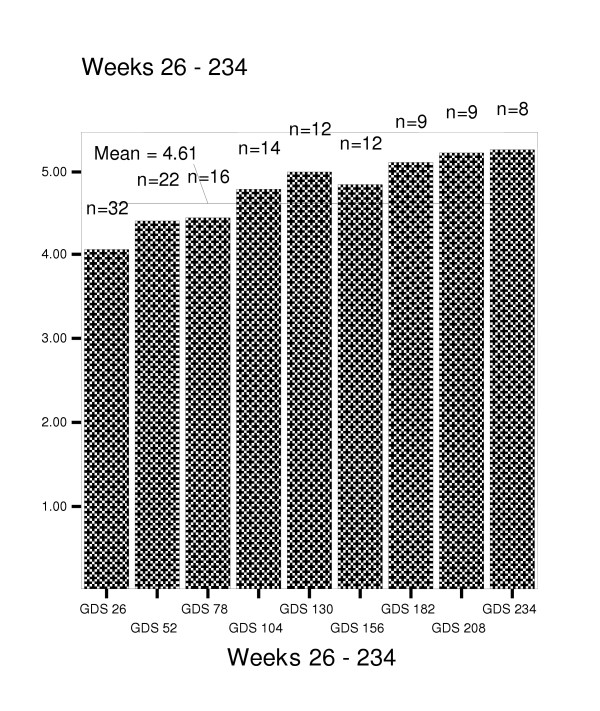
Mean scores on Geriatric Deterioration Scale for weeks 26–234.

**Figure 6:**
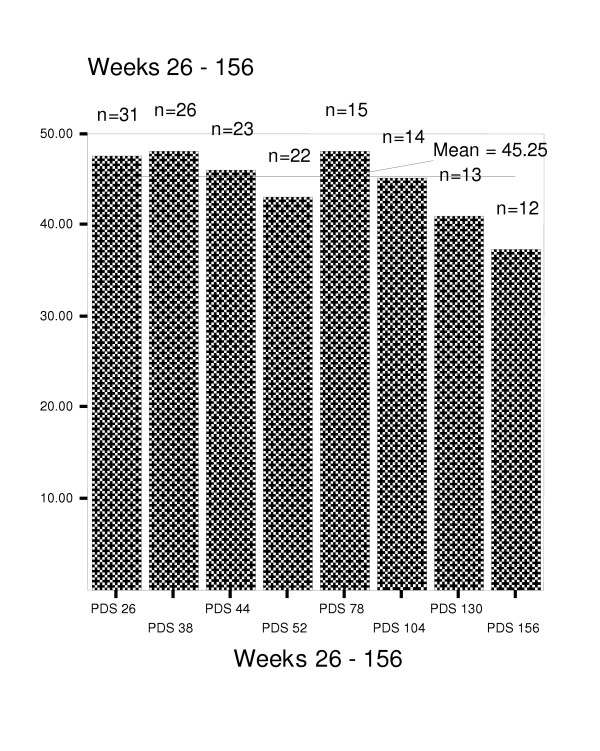
Mean scores on Progressive Deterioration Scale for weeks 26–156.

**Figure 7:**
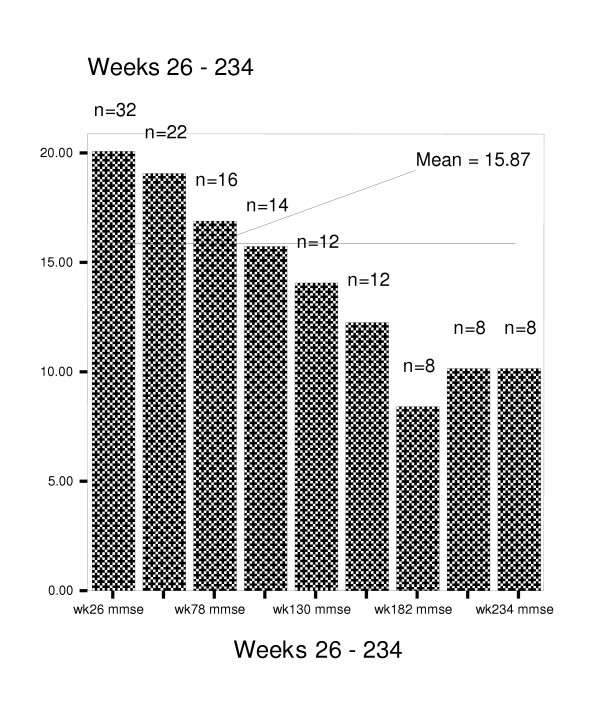
Mean scores on Mini-Mental Status Examination for weeks 26–234.

## Pre-publication history

The pre-publication history for this paper can be accessed here:



## References

[B1] Buckwalter K, Hall G, McBride AB, Austin JK (1996). Alzheimer's disease. Psychiatric Mental Health Nursing.

[B2] Beck C, Cody M, Souder E, Zhang M, Small W (2000). Dementia diagnostic guidelines Methodologies, results, and implementation costs. J Am Geriatr Soc.

[B3] Richards S, Hendrie H (1999). Diagnosis, management, and treatment of Alzheimer disease: a guide for the internist. Arch Intern Med.

[B4] Rogers S, Farlow M, Doody R, Mohs R, Friedhoff L, the Donepezil Study Group (1998). A 24-week, double-blind, placebo-controlled trial of donepezil in patients with Alzheimer's disease. Neurology.

[B5] Corey-Bloom J, Anand R, Veach J, for the ENA-713 B352 Study Group (1998). A randomized trial evaluating the efficacy and safety of ENA 713 (rivastigmine tartrate), a new acetylcholinesterase inhibitor, in patients with mild to moderately severe Alzheimer's disease. Int J Geriatr Psychopharmacol.

[B6] Raskind M, Peskind E, Wessel T, Yuan W, the Galantamine USA-1 Study Group (2000). Galantamine in AD, a 6-month randomized placebo-controlled trial with a 6-month extension. Neurology.

[B7] Doody R, Geldmacher D, Gordon B, Perdomo C, Pratt R, the Donepezil Study Group (2001). Open-label, multicenter, phase 3 extension study of the safety and efficacy of donepezil in patients with Alzheimer disease. Arch Neurol.

[B8] Lopez O, Becker J, Wisniewski S, Sazton J, Kaufer D, DeKosky S (2002). Cholinesterase inhibitor treatment alters the natural history of Alzheimer's disease. J Neurol Neurosurg Psychiatry.

[B9] Farlow M, Anand R, Messina J, Hartman R, Veach J (2000). A 52-week study of the efficacy of rivastigmine in patients with mild to moderately severe Alzheimer's disease. Eur Neurol.

[B10] Jacobs D, Sano M, Marder K, Bell K, Byslma F, Lafleche G, Albert M, Brandt J, Stern Y (1994). Age at onset of Alzheimer's disease: relation to pattern of cognitive dysfunction and rate of decline. Neurology.

[B11] Koss E, Edland S, Fillenbaum G, Mohs R, Clark C, Glasko D, Morris J (1996). Clinical and neuropyschological differences between patients with earlier and later onset of Alzheimer's disease: a CERAD analysis, part XII. Neurology.

[B12] Lucca U, Comelli M, Tettamanti M, Tiraboschi P, Spagnolli A (1993). Rate of progression and prognostic factors in Alzheimer's disease: a prospective study. J Am Geriatr Soc.

[B13] Mortimer J, Ebbitt B, Jun S, Finch M (1992). Predictors of cognitive and functional progression in patients with probable Alzheimer's disease. Neurology.

[B14] Mungas D, Reed B, Ellis W, Jagust W (2001). The effects of age on rate of progression of Alzheimer disease and dementia with associated cerebrovascular disease. Arch Neurol.

[B15] Seltzer B, Sherwin I (1983). A comparison of clinical features in early- and late-onset primary degenerative dementia. One entity or two?. Arch Neurol.

[B16] Wilson R, Gilley D, Bennett D, Beckett L, Evans D (2000). Person-specific paths of cognitive decline in Alzheimer's disease and their relation to age. Psychol Aging.

[B17] Stern Y, Tang M, Albert M, Brandt J, Jacobs D, Bell K, Marder K, Sano M, Devanand D, Albert S, Bylsma F, Tsai W (1997). Predicting time to nursing home care and death in individuals with Alzheimer disease. JAMA.

[B18] Woo J, Kim J, Lee J (1997). Age of onset and brain atrophy in Alzheimer's disease. Int Psychogeriatr.

[B19] Huff F, Growdon J, Corkin S, Rosen T (1987). Age at onset and rate of progression of Alzheimer's disease. J Am Geriatr Soc.

[B20] Bracco L, Gallato R, Grigoletto F, Lippi A, Lepore V, Bino G, Lazzaro M, Carella F, Piccolo J, Possilli C (1994). Factors affecting course and survival in Alzheimer's disease. A 9-year longitudinal study. Arch Neurol.

[B21] Boller F, Becker J, Holland A, Forbes M, Hood P, McGonigle-Gibson K (1991). Predictors of decline in Alzheimer's disease. Cortex.

[B22] Bowler J, Munoz D, Merskey H, Hachinski V (1998). Factors affecting the age of onset and rate of progression of Alzheimer's disease. J Neurol Neurosurg Psychiatry.

[B23] Doody RS, Massman P, Dunn JK (2001). A method for estimating progression rates in Alzheimer disease. Arch Neurol.

[B24] Drachman D, O'Donnell B, Lew R, Swearer J (1990). The prognosis inAlzheimer's disease. 'How far' rather than 'how fast' best predicts the course. Arch Neurol.

[B25] Stern R, Mohs R, Davidson M, Schmeidler J, Silverman J, Kramer-Ginsberg E, Searcey T, Bierer L, Davis K (1994). A longitudinal study of Alzheimer's disease: measurement, rate, and predictors of cognitive deterioration. Am J Psychiatry.

